# Selenium Valence-to-Core X-ray Emission Spectroscopy and Kβ HERFD X-ray Absorption Spectroscopy as Complementary Probes of Chemical and Electronic Structure

**DOI:** 10.1021/acs.inorgchem.1c02802

**Published:** 2022-02-03

**Authors:** Justin T. Henthorn, Serena DeBeer

**Affiliations:** Max Planck Institute for Chemical Energy Conversion, Stiftstrasse 34-36, D-45470 Mülheim an der Ruhr, Germany

## Abstract

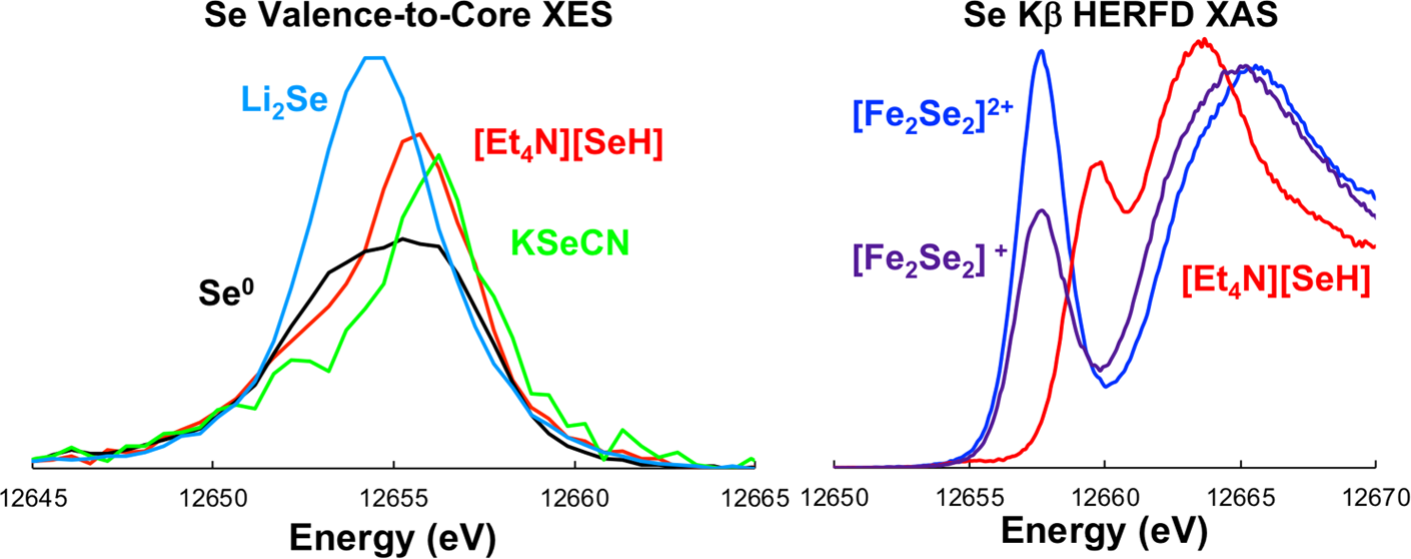

Selenium X-ray absorption spectroscopy (XAS) has found widespread use in investigations of Se-containing materials, geochemical processes, and biologically active sites. In contrast to sulfur Kβ X-ray emission spectroscopy (XES), which has been found to contain electronic and structural information complementary to S XAS, Se Kβ XES remains comparatively underexplored. Herein, we present the first Se Valence-to-Core (VtC) XES studies of reduced Se-containing compounds and FeSe dimers. Se VtC XES is found to be sensitive to changes in covalent Se bonding interactions (Se–Se/Se–C/Se–H bonding) while being relatively insensitive to changes in Fe oxidation states as selenide bridges in FeSe dimers ([Fe_2_Se_2_]^2+^ vs [Fe_2_Se_2_]^+^). In contrast, Se Kβ HERFD XAS is demonstrated to be quite sensitive to changes in the Fe oxidation state with Se Kβ HERFD XAS demonstrating experimental resolution equivalent to Kα HERFD XAS. Additionally, computational studies reveal both Se VtC XES and XAS to be sensitive to selenium protonation in FeSe complexes.

## Abstract 2

Selenium is a trace element that plays vital roles in biological and geochemical cycles, energy storage, photovoltaics, and nanomaterials. Herein, selenium Valence-to-Core X-ray emission spectroscopy is explored as a new method of probing the chemical and electronic structure in selenium-containing compounds, demonstrating sensitivity to selenium bonding interactions. When paired with high-resolution Se X-ray absorption spectroscopy (HERFD XAS), these two methods have the potential to reveal greater insight into protonation and redox changes of Se-substituted FeS clusters.

## 1 Introduction

Selenium is an essential trace element that plays pivotal functions in biological^1−3^ and environmental sciences.^4^ Additionally, selenium is a semiconductor with applications in the fields of nanoscience^5,6^ and photovoltaics.^7−9^ Other applications include energy storage^10,11^ and glass manufacturing. Across many of these applications, there is great utility for an element-selective spectroscopy to better understand the electronic and chemical structure of the relevant selenium species. ^77^Se NMR has proven useful in characterizing the chemical environment of Se;^12−19^ however, the requirement of diamagnetic samples and solution-based measurements limits its broad applicability, as well as constrains its potential for *in situ* experiments. Se X-ray absorption spectroscopy has been implemented in some of these applications.^20−25^ Se XAS is directly analogous to S XAS, which has recently been coupled with S XES as a complementary probe of electronic and chemical structure.^26,27^ In comparison, Se XES remains largely unexplored. Herein, we report the first Se Kβ XES study of reduced Se compounds, including biologically relevant [Fe_2_Se_2_]^*n*+^ complexes.

## 2 Experimental Methods

### 2.1 Sample Preparation

Li_2_Se and [Et_4_N][SeH] were synthesized following published procedures.^100,101^ KSeCN and gray (hexagonal) elemental Se (Se^0^) were purchased from Sigma and used as received. The β-diketiminate-supported iron dimer complexes L_2_Fe_2_Se_2_ and [K(THF)_6_][L_2_Fe_2_Se_2_]·2THF (L = {HC[C(CH_3_)N-(2,6-^*i*^Pr_2_C_6_H_3_)]_2_}^1–^) were prepared according to literature methods.^28^ All synchrotron measurements were performed on solid samples diluted in BN (Sigma, dried under vacuum for 2 h at 120 °C) to ∼1% Se by weight and finely ground with mortar and pestle, except KSeCN that was measured as a frozen 1 mM solution in distilled H_2_O. The solid samples were packed into 1 mm thick aluminum spacers and sealed with a 38 μm Kapton tape, while the KSeCN sample was prepared in a Delrin pinhole solution cell with a 38 μm Kapton tape window and frozen in liquid nitrogen.

### 2.2 Data Collection and Processing

All presented data were measured at beamline ID-26 of the European Synchrotron Radiation Facility (ESRF) with the storage ring operating at 6 GeV and injection currents of 90 mA in a 16-bunch filling mode. A Si(3 1 1) double-crystal monochromator was used upstream for incident energy selection. The monochromatic incident energy was calibrated to the absorption maximum of gray elemental selenium (12 659.8 eV). The beam size was 500 × 100 (horizontal × vertical) μm^2^, providing a flux density of ∼1 × 10^12^ ph/s. Selenium X-ray absorption spectra were measured simultaneously in total fluorescence yield (TFY) and Kβ_1_ HERFD detection modes. For the emission measurements, a 1 m radius Johann-type XES spectrometer was used, equipped with four spherically bent Ge(8 8 0) analyzer crystals aligned on intersecting Rowland circles. The XES spectrometer was internally calibrated using the emission lines of gray elemental selenium (Kβ_1_ = 12 492.6 eV; Kβ_3_ = 12 498.0 eV; Kβ_2_ = 12 655.1 eV). Kβ_1_-detected XAS and Kβ XES data collection was performed using a dead-time corrected silicon drift diode detector (Ketek) aligned on the Rowland circle. Attenuation of the fluorescence signal was reduced by placing a He-filled flight path between the sample, the analyzer crystals, and the detector. Measurements were performed in a liquid helium flow cryostat maintained at ∼20 K.

Se Kβ HERFD XAS was collected over an energy range of 12 640–12 700 eV in 0.1 eV steps. The incident energy was set to 13 000 eV to collect nonresonant Se Kβ XES. Emission scans were collected varying the scan parameters in three different ranges: 12 485–12 510, 12 505–12 640, and 12 640–12 670 eV using energy step sizes of 0.5, 1.0, and 0.5 eV, respectively. The total energy resolution was estimated to be ∼1.5 eV based on the full width at half-maximum of the elastic peaks. The width of the elastic peak (Δ*E*) results from a convolution of the spectrometer and monochromator resolution.

Radiation damage studies were performed on each individual sample by collecting successive fast energy range XAS scans (10 s/scan) at a single spot on the sample, averaging across multiple spots to increase the signal-to-noise ratio and better observe spectral changes indicative of damage. All samples eventually revealed damage in the beam. The maximum exposure time per spot to collect undamaged data on all samples was 30–120 s (applied to both XAS and XES measurements). The presented data comprises an average of ∼10 XAS and ∼6 XES scans for each compound (each scan corresponding to a fresh spot on the sample). Total collection times for XAS and XES measurements were approximately 20 and 60 min per sample, respectively. The long XES measurement time (relative to the short exposure time per spot) is due to slow motor stepping across the large spectrometer energy window (ca. 175 eV). Fast shutters were used to protect the sample during motor stepping, maintaining the maximum exposure time threshold per spot for undamaged data.

Individual scans showing no evidence of radiation damage were first averaged with the PyMCA software package.^29^ XAS spectra were background corrected and normalized by setting the edge jump to 1. Experimental spectra were fit in an energy range from 12 640 to 12 670 eV as a sum of 1–3 pseudo-Voigt functions in the pre-edge region and 3–5 pure Gaussian functions in the edge region using an iterative least-squares Matlab script. In all cases, the fits converged to pre-edge pseudo-Voigt functions with ≤30% Lorentzian composition. All energies and areas reported are from the corresponding fits, with pre-edge areas taken as the sum of the areas of the pre-edge functions multiplied by 100. Edge energies are reported as the white line maxima and pre-edge energies are reported as intensity-weighted average of the pre-edge fitting functions.

XES spectra were normalized to the integrated intensity of the Kβ_1,3_ mainlines (12 490–12 510 eV). The intensities and energy positions of Kβ_2_ XES data were extracted by modeling the experimental line shapes with pseudo-Voigt functions using an iterative least-squares Matlab script. All energies (intensity-weighted averages) and areas are reported from the corresponding fits, with areas taken as the sum of the areas of the fitting functions multiplied by 100 000.

### 2.3 Computational Details

All geometry optimizations and ground-state and TDDFT^30−32^ calculations were executed using ORCA^33,34^ version 4.1. Computations were performed using the hybrid TPSSh^35,36^ functional with the D3BJ^37,38^ dispersion correction and CPCM^39−41^ solvation model. The ZORA^42,43^ relativistic approximation was used and employed the relativistically contracted def2 Ahlrichs^44,45^ basis set. A triple-ζ ZORA-def2-TZVP basis set was used for all Se, S, Fe, and N atoms, while a double-ζ def2-SVP basis set was used for all other atoms. The RIJCOSX^46,47^ approximation was used to speed up Coulombic and exchange integrals. For the Fe dimer complexes discussed in this work, appropriate antiferromagnetic ground states were achieved starting from a “high spin” ferromagnetic solution and employing the spinflip keyword to access the broken symmetry solution. In the case of Li_2_Se, a [Li_8_Se]^6+^ model was employed using coordinates from a recently reported crystal structure (ICSD 1684308). Additionally, a geometry-optimized linear HSe(Se)_6_SeH compound was employed as a model of Se^0^ and the VtC XES spectrum was calculated from the two central Se atoms. Calculations for all other compounds employed geometry-optimized molecular models.

Computational core-level spectroscopy for XES was carried out by a ground-state DFT procedure, where transition energies are based on energy differences between one-electron Kohn–Sham orbitals, as previously reported.^48^ The hybrid TDDFT calculations were performed using 100–400 roots (depending on the system) to ensure the maximum of the rising edge was calculated. Se XAS and XES spectra were plotted with applied broadenings of 2.0 and 5.0 eV (fwhm) and shifted by constant values of −77.3 and −77.0 eV, respectively. Calculated Se XAS spectra were normalized by dividing by 5.3 to reproduce the pre-edge intensities observed experimentally for the [Fe_2_Se_2_]^*n*+^ complexes (*n* = 1, 2), and calculated Se XES spectra were normalized by dividing by 120 000 to reproduce the intensities observed experimentally for the organoselenide series. Sample input files for calculations, as well as coordinates for all model systems, can be found in the Supporting Information.

## 3 Results and Discussion

### 3.1 Se Kβ XES

**Figure 1:**
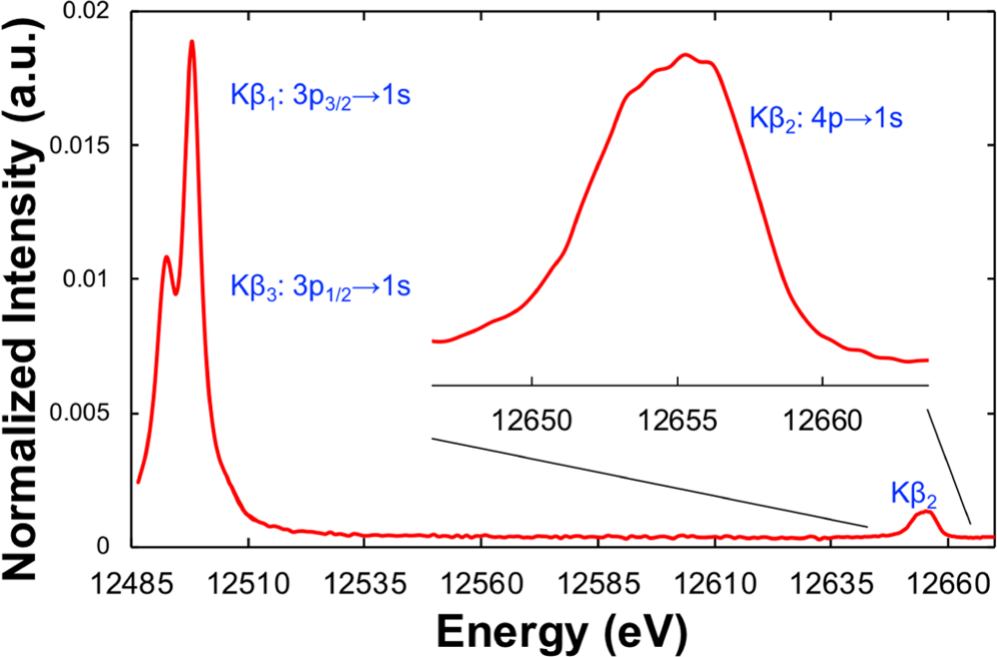
Full Se Kβ XES spectrum of gray elemental selenium, including the Kβ_1,3_ mainline and Kβ_2_ valence-to-core regions. Inset shows the Kβ_2_ region in greater detail.

Se Kβ XES corresponds to the decay processes of Se 3p → 1s and 4p → 1s emission events (Figure 1). The 3p → 1s emission event is dominated by 3p spin–orbit coupling (SOC), splitting into Kβ_1_ (3p_3/2_ → 1s) and Kβ_3_ (3p_1/2_ → 1s) transitions. The 4p → 1s emission Kβ_2_ is ca. 150 eV higher in energy and represents the valence-to-core transitions. As such, the Kβ_2_ is dominated by the bonding interactions of the Se photoabsorber.

### 3.2 Se Kβ_1,3_ Mainline

**Figure 2:**
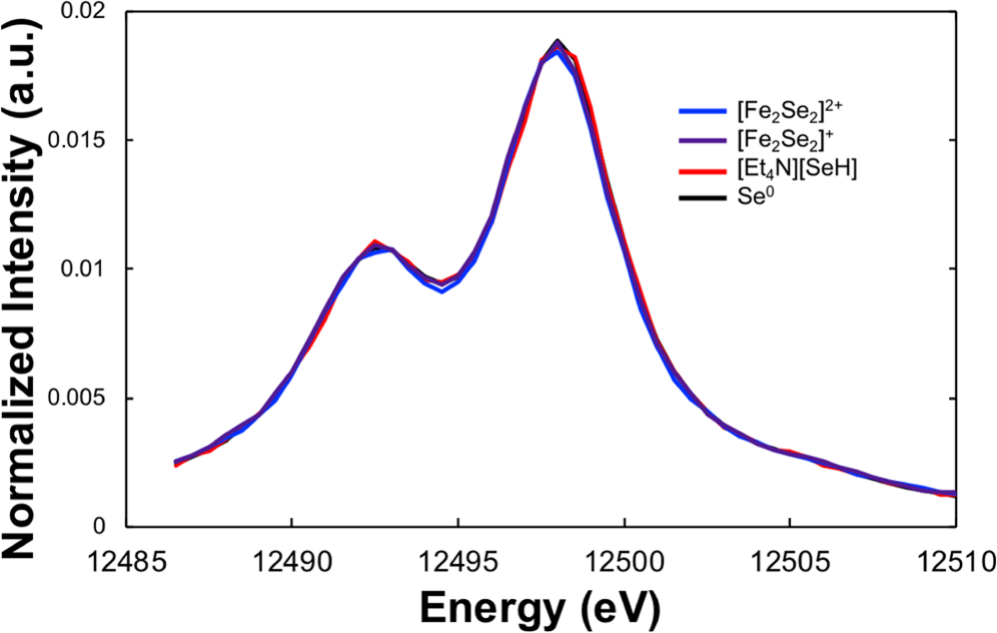
Se Kβ mainline (Kβ_1,3_) comparison of four reduced selenium compounds: [Fe_2_Se_2_]^2+^ (blue), [Fe_2_Se_2_]^+^ (purple), [Et_4_N][SeH] (red), and elemental selenium (black).

The Kβ_1,3_ region of the Kβ XES spectrum is split by the 3p core-hole SOC to give a spectral shape similar to S Kα XES,^49^ albeit with much larger splitting of the Kβ_1,3_ lines (∼5.1 eV for Se Kβ_1,3_ compared to ∼1.2 eV for S Kα_1,2_), consistent with the larger SOC of Se relative to S. The analogy of second-row transition-metal Kβ XES to first-row transition-metal Kα XES has previously been demonstrated.^50^ Analogous to S Kα XES, the Se Kβ_1,3_ mainline region is anticipated to be sensitive to the relative oxidation state of selenium, though comparatively less sensitive than S Kα XES due to greater shielding of the nucleus by the Se 3d orbitals. Figure 2 shows a comparison of the Se Kβ_1,3_ mainline spectra of several reduced forms of Se: [Fe_2_Se_2_]^*n*+^ complexes (*n* = 1, 2) and hydroselenide (all formally Se^II–^) as well as elemental selenium (Se^0^). Within the resolution of the experiment, all four spectra are identical, consistent with DFT calculations (one-electron method, Figure S1) that predict at most a shift of 100 meV in the 3p to 1s transition energies. Even DFT calculations for the more oxidized compounds SeO_3_^2–^ and SeO_4_^2–^ (Se^IV^ and Se^VI^, respectively) suggest only modest shifts in the 3p to 1s transition energy, with a maximum difference of ∼0.3 eV (from Se^0^ to SeO_4_^2–^, Figure S2). Overall, these results reveal the Se Kβ_1,3_ mainline to be much less sensitive to the formal oxidation state than S Kα XES (Δ*E* = 1.43 eV^49^ from Na_2_SO_4_ to ZnS).

### 3.3 Se Kβ_2_ Valence-to-Core

**Figure 3:**
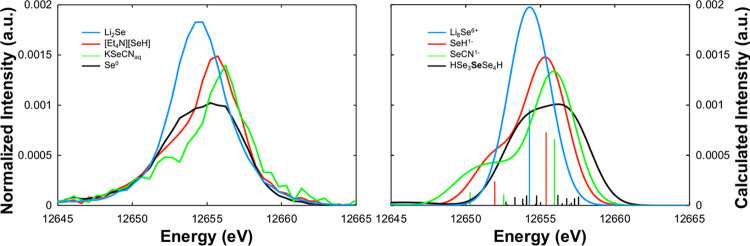
Background-subtracted experimental (left) and DFT-calculated (right) Se VtC XES spectra of Li_2_Se (light blue), [Et_4_N][SeH] (red), KSeCN (green), and elemental selenium (black). Individual calculated transitions are shown as vertical sticks, while line-broadened spectra are shown as curves.

The Se Kβ_2_ XES spectra (hereafter referred to as VtC XES) of Li_2_Se, [Et_4_N][SeH], KSeCN, and gray elemental selenium (Se^0^) are presented in Figure 3. It is immediately clear that there are dramatic and distinct changes across the series of compounds. Starting with the ionic Li_2_Se (Figure 3, blue), the VtC XES spectrum reveals an intense symmetric feature centered at 12 655 eV. Addition of a single covalent bond to selenium results in a distinctly asymmetric VtC XES spectrum, as evidenced by [Et_4_N][SeH] (Figure 3, red) and KSeCN (Figure 3, green), with the more intense feature shifted to higher energy (ca. 12 656 and 12 657 eV, respectively) and a significant shoulder shifted to lower energy (ca. 12 653 eV). Finally, the fully covalent Se^0^ VtC XES spectrum (Figure 3, black) reveals a broadened, slightly asymmetric emission feature of lower intensity centered around 12 655 eV. These spectra can easily be understood from a simplistic molecular orbital (MO) diagram picture, as detailed below.

Starting at the atomic limit of selenide (Se^2–^), the three 4p donor orbitals (4p_*x*_, 4p_*y*_, and 4p_*z*_) are degenerate, resulting in a single, narrow VtC XES transition in Li_2_Se (Figure S3, right). Upon protonation to form hydroselenide (SeH^1–^), the more positive charge on Se (2– to 1−) will result in a stabilization of the 1s orbital (due to increased *Z*_eff_), resulting in a shift of the 4p-to-1s transition to higher energy (Figure S3, left). Additionally, mixing of a Se 4p orbital with the H 1s orbital results in a bonding MO that is filled and stabilized relative to the Se 4p energy level, as well as an unfilled antibonding MO that is destabilized relative to the Se 4p energy level. Thus, the Se VtC XES spectrum will have two main transitions, one shifted to lower energy (relative to the Se^2–^ spectrum) arising from the Se–H bonding interaction, and the second transition shifted to higher energy (relative to the Se^2–^ spectrum) arising from the two remaining nonbonding Se 4p orbitals. The relative intensity of the nonbonding transition will be ∼2/3 the intensity of the Se^2–^ spectrum, while the Se–H transition will be less than half of the nonbonding transition (as the overlap of the bonding MO with the Se 1s orbital has decreased relative to the nonbonding Se 4p atomic-like orbital). A similar MO picture can be drawn for SeCN^1–^ (Figure S4), resulting in qualitatively similar transitions arising from the nonbonding Se 4p orbitals and Se–C_σ_ bonding orbitals, with the expectation that the more electronegative CN group will further stabilize the Se 1s orbital resulting in a shift of the Se 4p-to-1s transition to even higher energy, and the stronger Se–C bond will result in a shift to lower energy of the Se–C_σ_ bonding transitions. Additionally, the Se p orbital can mix with both the CN_σ_ (p-parentage) and CN_σ*_ (s-parentage) orbitals, giving rise to two bonding transitions in the Se VtC XES. These simple pen-and-paper calculations are borne out by our DFT calculations (Figure 3), which well-match the experimental results.

For elemental selenium, the simple MO picture becomes slightly more complicated due its oligomeric nature (see Figure S5 for MO diagram of HSeSeSeH as a simple model). However, we can assume a set of filled Se–Se bonding orbitals (and corresponding empty antibonding orbitals), as well as a set of largely nonbonding orbitals, giving rise to two broad regions in the Se VtC XES spectrum, matching well the experimentally observed asymmetric Se^0^ VtC XES spectrum (Figure 3, black). DFT calculations of a similar model system (see Section 2.3) well reproduce the experimental spectrum, evidencing the more complicated MO picture due to a large amount of covalent mixing with neighboring Se centers in the oligomeric structure (compare calculated individual transitions shown as black vertical sticks in Figure 3).

Quantitatively, we can also observe that the overall intensity (total area, determined from fitting the experimental spectra, see Experimental Section) of the Se VtC XES spectra decreases in the order Se^2–^ > HSe^1–^ > NCSe^1–^ > Se^0^, corresponding to increasing covalent bonding interactions (Table 1, Figures S6–S8). This inverse correlation of Se VtC XES intensity to bonding covalency is consistent with the mechanism of VtC XES intensity, which is proportional to the transition dipole integral (the “allowedness” of the transition), the donor–acceptor orbital overlap (Se 4p/1s), and the number of electrons in the donor orbitals. As the 4p → 1s transition is formally dipole allowed, the bulk of the intensity from these closed-shell main-group Se compounds arises from the 4p/1s orbital overlap. At the ionic limit of Li_2_Se, the Se 4p orbitals are strongly localized to the Se center, maximizing the Se 4p–Se 1s orbital overlap. As covalent bonds are formed between the photoabsorbing selenium and other atoms (H, C, Se), the generated bonding MOs of Se 4p-parentage are less localized to the Se, resulting in decreased Se 4p/1s overlap and thus decreasing Se VtC XES spectral intensities. In the case of Se^0^, the formal loss of electrons relative to the selenide compounds results in further contraction of the Se orbitals, additionally contributing to the decrease in VtC intensity.

**Table 1:** Se VtC XES Energies and Areas

sample	energy^a^ (eV)	area
Li_2_Se	12 654.54	960
[Et_4_N][SeH]	12 654.90	820
KSeCN	12 655.25	740
Se^0^	12 654.42	730
[Fe_2_Se_2_]^2+^	12 654.76	890
[Fe_2_Se_2_]^+^	12 654.70	930

a Area-weighted average of fitting functions.

**Figure 4:**
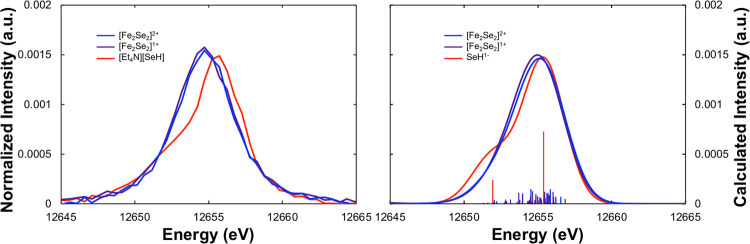
Background-subtracted experimental (left) and DFT-calculated (right) Se VtC XES spectra of [Fe_2_Se_2_]^2+^ (blue), [Fe_2_Se_2_]^+^ (purple), and [Et_4_N][SeH] (red). Individual calculated transitions are shown as vertical sticks, while line-broadened spectra are shown as curves.

Turning now to FeSe clusters, we examine the effects of the Fe oxidation state on the Se VtC XES spectra of a μ-Se bridge in β-diketiminate-supported [Fe_2_Se_2_]^*n*+^ complexes (*n* = 1, 2). From our simple MO picture developed through analysis of the Se VtC XES spectra above, we can qualitatively predict that the Se VtC XES spectra of the FeSe dimer complexes should lie somewhere between the Li_2_Se and Se^0^ spectra, both in terms of lineshape and overall intensity, as the Fe–Se bonding interactions are assumed to be more covalent than the highly ionic Li_2_Se, but less covalent than elemental selenium. Indeed, as experimentally observed in Figure 4, the spectra of the FeSe dimers are intermediate in intensity between Li_2_Se and Se^0^, while lacking the large asymmetry present in [Et_4_N][SeH] and KSeCN, consistent with two Fe–Se bonding interactions. Interestingly, there is little obvious change in the Se VtC XES spectrum upon one-electron redox of the dimer. DFT calculations reproduce this observation, with the mixed-valent dimer [Fe_2_Se_2_]^+^ having only slightly increased intensity relative to the diferric complex [Fe_2_Se_2_]^2+^. Experimentally, the [Fe_2_Se_2_]^+^ complex also exhibits a slightly more intense Se VtC spectrum than the [Fe_2_Se_2_]^2+^ complex (area = 930 for [Fe_2_Se_2_]^+^ vs 890 for [Fe_2_Se_2_]^2+^). This slight increase in intensity for [Fe_2_Se_2_]^+^ relative to [Fe_2_Se_2_]^2+^ is again consistent with the decreased covalency of the Fe–Se bonds in the reduced dimer.

### 3.4 Kβ HERFD XAS

To maximize the information content available through measuring Se Kβ XES, we also explored Se Kβ high-energy-resolution fluorescence detection XAS (or Kβ HERFD XAS) using the same experimental setup. Analogous to Se Kα HERFD XAS^21,22,51^ (which uses the Kα_1_ emission), Se Kβ HERFD XAS selectively measures the Kβ_1_ emission maximum across the Se absorption edge. In the case of Kα HERFD XAS, experimental resolution is improved approximately two-fold compared to partial fluorescence yield measurements due to the significantly longer-lived 2p core-hole lifetime relative to the 1s core-hole lifetime. Moving to Kβ HERFD XAS, the even longer-lived 3p core-hole lifetime^52^ similarly could further improve the spectral resolution relative to the standard PFY measurement; however, additional multielectron decay pathways (*i.e*., Coster–Krönig transitions) could limit any improvement in resolution relative to Kα HERFD XAS. Experimentally, we find that Kα and Kβ HERFD XAS exhibit nearly identical spectral resolution, as shown in Figure S11. Thus, the intrinsic increased resolution due to the longer 3p core-hole lifetime is only moderately diminished via additional decay pathways, resulting in an overall similar spectral resolution to the Kα HERFD measurement. However, the Kβ emission is approximately an order of magnitude lower in intensity than the Kα emission, and thus Kβ HERFD XAS will likely require longer collection times to achieve the same signal-to-noise ratio as Kα HERFD XAS, particularly with low-concentration samples.

**Figure 5:**
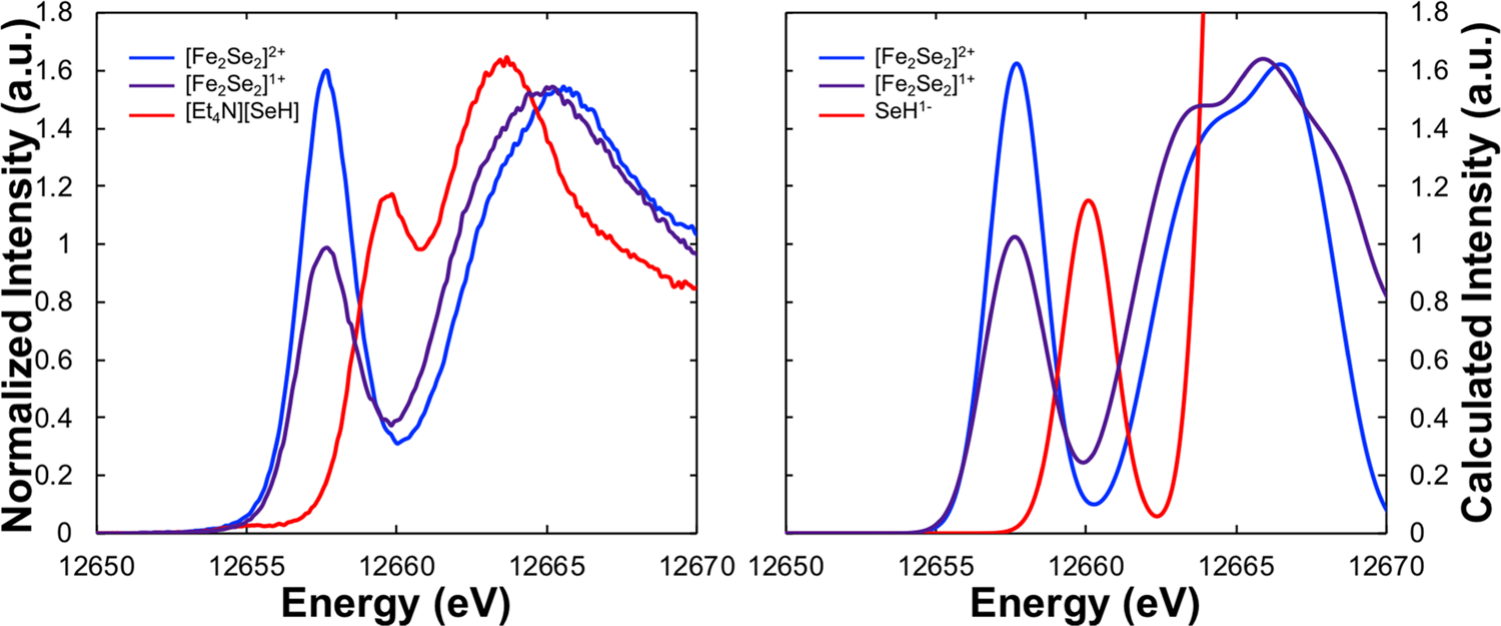
Experimental (left) and TDDFT-calculated (right) Kβ_1_ HERFD XAS spectra of [Fe_2_Se_2_]^2+^ (blue), [Fe_2_Se_2_]^+^ (purple), and [SeH]^1–^ (red).

The Kβ HERFD XAS spectra of the diferric [Fe_2_Se_2_]^2+^ and mixed-valent [Fe_2_Se_2_]^+^ complexes are presented in Figure 5. As anticipated, the diferric complex [Fe_2_Se_2_]^2+^ reveals an intense pre-edge feature (area = 385) around 12 653 eV, consistent with the more covalent Fe–Se interactions and high d-hole count (10) in the oxidized dimer core (Table 2). Upon one-electron reduction to the mixed-valent [Fe_2_Se_2_]^+^ complex, the pre-edge feature decreases in intensity (area = 260) and the rising edge shifts to lower energy, with the white line maximum decreasing from 12 655.56 eV in [Fe_2_Se_2_]^2+^ to 12 664.99 eV in [Fe_2_Se_2_]^+^, a net change of −0.57 eV. The decrease in pre-edge intensity and shift to lower energy of the rising edge are both consistent with a less covalent Fe–Se interaction in the reduced dimer (as well as a decrease in d-hole count to 9) and overall destabilization of the 1s Se orbital due to a lower *Z*_eff_. We note that the pre-edge intensity of the diferric complex [Fe_2_Se_2_]^2+^ (area = 385) is consistent with the previously measured Kα HERFD XAS spectra of the diferric [^*n*^Et_4_N]_2_[Fe_2_Se_2_(SPh)_4_] (370) as well as the Se_2B_ spectrum of resting-state FeMoco in nitrogenase (385 ± 20), which has been assigned as an antiferromagnetically coupled diferric site.^21^ The present results further support this assignment.

**Table 2:** Se Kβ_1_ HERFD XAS Edge Energies, Pre-edge Energies and Areas, and Relative Fe 3d-Manifold Energies

	edge	pre-edge		
sample	energy (eV)	energy (eV)	area	|δ_d_|^a^ (eV)
[Fe_2_Se_2_]^2+^	12 665.56	12 657.70	385	7.86
[Fe_2_Se_2_]^+^	12 664.99	12 657.73	260	7.26
[Et_4_N][SeH]	12 663.57	12 659.47	260	
Se^0^	12 667.72	12 660.12	740	

a |δ_d_| = |(pre-edge energy) – (edge energy)|.

The dramatic change in pre-edge intensity in the Kβ HERFD XAS spectrum upon one-electron redox of the [Fe_2_Se_2_]^*n*+^ core, in contrast to the negligible intensity difference observed in the Kβ XES spectra, requires additional investigation and discussion. We turn now to DFT calculations to better elucidate the disparity.

### 3.5 Theoretical Investigations of [Fe_2_SSe]^*n*+^ and [Fe_2_S(SeH)]^*n*+^ Systems

To better understand the dramatic differences in the effects of oxidation-state changes between Se VtC XES (where there is minimal effect) and XAS (where there is significant effect) in our measured [Fe_2_Se_2_]^*n*+^ dimers, we have computationally investigated a similar set of fictitious [Fe_2_SSe(SMe)_4_]^*n*−^ (*n* = 2, 3) complexes. This fictitious dimer system exhibits nearly identical Se VtC XES and XAS spectra to the experimentally investigated compounds (Figure S12), demonstrating the minimal perturbative effect of substituting the bridging (S vs Se) and terminal (thiolate vs nacnac) ligands, and the accompanying geometric changes, on the properties of the Se VtC XES and XAS spectra. Analysis of the Se 4p orbitals via the intrinsic atom orbitals and intrinsic bonding orbitals (IAOIBO) method^53^ reveals the Fe–Se bonding orbitals are on average 75.1% Se composition (Löwdin) in the oxidized dimer and 76.4% Se composition in the mixed-valent dimer, while the remaining nonbonding orbitals are 85.6 and 86.6% Se, respectively (Figures S13 and S14). These MO analyses are consistent with the experimental Se VtC XES spectra of the [Fe_2_Se_2_]^*n*+^ complexes (*n* = 1, 2), wherein minimal change in Se composition of the Fe–Se bonding and Se nonbonding MOs upon one-electron redox results in a correspondingly minimal change in the VtC spectra.

In contrast, analysis of the Fe 3d manifold reveals a similarly small change (in absolute terms) of the Se contributions to the MOs upon one-electron redox, from an average of 6.44% Se p character in the oxidized dimer to 5.28% (Δ = −1.16%) in the mixed-valent dimer, well mirroring the change in the Se orbital contribution to the Fe–Se bonding MOs upon one-electron redox from 75.1 to 76.4% (Δ = +1.3%). In the case of the Fe–Se bonding MOs, the change in the Se orbital contribution has a negligible effect on the Se VtC XES spectra as the Se contribution (and by extension the Se 4p/1s orbital overlap) only changes in relative terms by ∼2%; however, the same absolute magnitude change (ca. 1%) in Se orbital contribution to the Fe 3d manifold results in a more dramatic change in the XAS pre-edge intensity, as the relative change in the Se orbital contribution is more substantial in the Fe 3d-manifold MOs (ca. 20%). This implies that the significant intensity of the XAS pre-edge feature in the diferric complex is dominated by the large number of acceptor orbitals (10 half-filled Fe 3d orbitals) rather than a large Fe/Se orbital overlap, and thus the large decrease in XAS pre-edge intensity upon one-electron reduction to the mixed-valent dimer is driven less by the decrease in the number of acceptor orbitals (9 down from 10, a factor of 0.9) and more by the large relative decrease in the Se p orbital contribution (5.28% down from 6.44%, a factor of 0.82) and by extension of the Se 4p/1s overlap. Indeed, the product of these two ratios (0.9 × 0.82 = 0.739) well approximates the decrease in pre-edge intensity experimentally observed upon one-electron reduction (260/385 = 0.675). Overall, it is the relatively ionic nature of these orbitals that minimizes the effects of oxidation-state changes in the Se VtC XES spectra, in contrast to the large changes observed in the corresponding Se XAS spectra.

**Figure 6:**
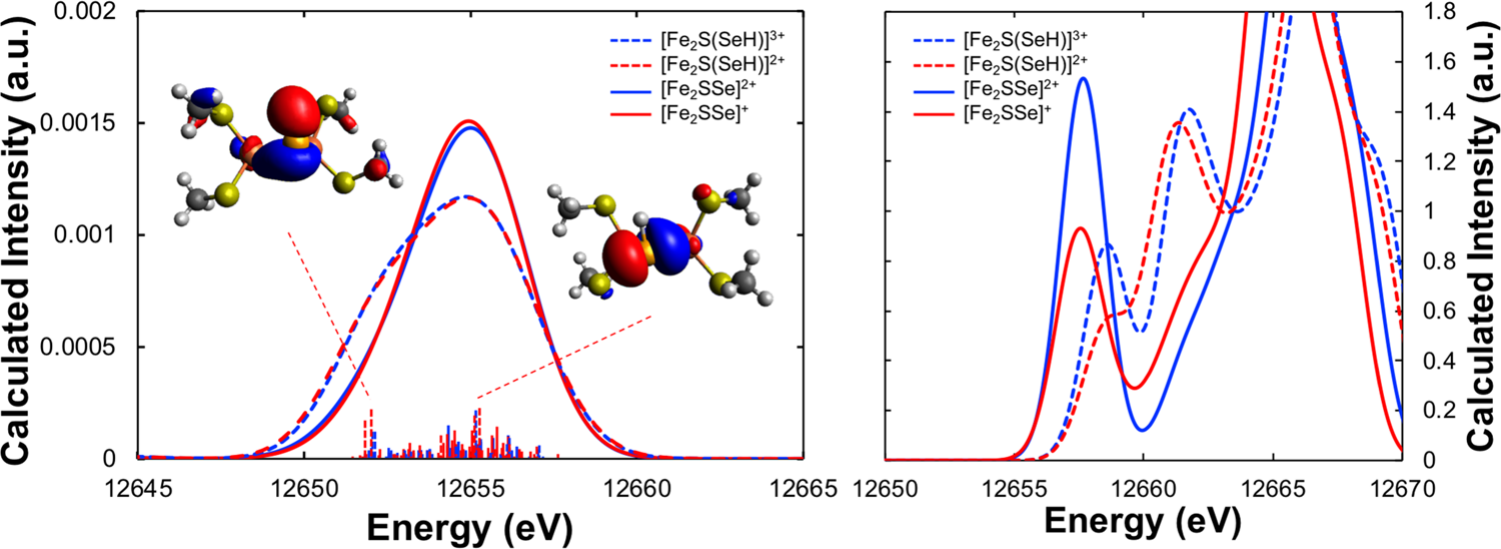
Calculated Se VtC XES (left) and XAS (right) spectra of fictitious [Fe_2_SSe]^2+^ (solid blue), [Fe_2_SSe]^+^ (solid red), [Fe_2_S(SeH)]^3+^ (dashed blue), and [Fe_2_S(SeH)]^2+^ (dashed red). Individual transitions are shown as vertical sticks. Inset shows MOs corresponding to the Fe–Se_σ_ donor transition and the Se–H_σ_ donor transition for the [Fe_2_S(SeH)]^2+^ complex.

While Se VtC XES shows little sensitivity to the Fe oxidation state in FeSe complexes, we have shown experimentally that it is sensitive to selenium protonation (see Section 3.3). Here, we further investigate computationally the utility of Se VtC XES and XAS as probes of selenium protonation in FeSe complexes through protonation of our fictitious [Fe_2_SSe]^*n*+^ models to yield the corresponding [Fe_2_S(SeH)]^(*n*+1)+^ complexes. In the VtC XES spectra (Figure 6), protonation of the bridging selenide yields new emission features arising from the Se–H_σ_ donor orbital, which appear at lower energy (ca. 12 653 eV) concomitant with the loss of intensity at higher energy (ca. 12 655 eV). These changes are consistent with the loss of the formal Se lone-pair transition upon protonation. Similar to the unprotonated system, the calculated Se VtC XES spectra reveal no sensitivity to one-electron redox, indicating VtC XES to be a more selective probe of selenium protonation (*vide infra*). In the XAS spectrum, protonation of the selenide bridge results in a decrease in the pre-edge feature and the appearance of a new absorption feature in the rising edge assigned to the Se 1s → Se–H_σ*_ transition. Similar trends in the Fe oxidation state occur with the [Fe_2_S(SeH)]^(*n*+1)+^ series, with decreasing pre-edge intensity with decreasing Fe oxidation state, as well as shifts in the rising edge to lower energy with decreasing oxidation state. Additionally, the Se–H_σ*_ transition also shifts to lower energy with decreasing Fe oxidation state, consistent with destabilization of the Se 1s orbital due to a higher *Z*_eff_. These results reveal that both VtC XES and XAS are sensitive to Se protonation in FeSe dimers, while only XAS is sensitive to changes in redox levels. As protonation often accompanies one-electron reduction in biological cofactors (proton-coupled electron transfer, PCET),^54,55^ Se XES would more clearly evidence a protonation event regardless of any redox change, while Se XAS would reveal the convolution of the protonation event and the redox change, potentially resulting in the protonation event being obscured. For this hypothetical PCET event, the XAS experiment may be difficult to interpret in isolation, but combined with XES the two effects could be more easily deconvoluted.

## 4 Conclusions

Through investigation of a series of reduced Se compounds, we have demonstrated the sensitivity of Se VtC XES to changes in the covalent bonding interactions of the photoabsorbing Se center. The observed experimental changes can easily be rationalized through simple MO analyses and are well-reproduced through DFT calculations. The Se Kβ_1,3_ mainline region reveals negligible changes in the reduced forms of Se investigated in this study (Se^2–^ and Se^0^), which are also well-matched by simple DFT calculations. Se VtC XES spectra of [Fe_2_Se_2_]^*n*+^ dimers revealed minor sensitivity to changes in the Fe oxidation state, in contrast to the high sensitivity of Se Kβ HERFD XAS. Computational analyses reveal the source of these contrasting sensitivities arises from the relatively ionic nature of the Fe–Se bonding interactions. Additionally, the sensitivity of Se VtC XES to protonation suggests a combined Se VtC XES/Kβ HERFD XAS approach could be a powerful tool in elucidating protonation/alkylation of Se within FeSe clusters, most notably in Se-substituted nitrogenase.^56,57^ Furthermore, we have demonstrated that Se Kβ HERFD XAS exhibits spectral resolution matching Se Kα HERFD XAS, and thus a single experimental setup allows access to complementary Se VtC XES and HERFD XAS measurements. Future studies will explore resonant measurements as a means of enhancing sensitivity to changes in Se chemical and electronic structure—analogous to recent Fe resonant X-ray emission spectroscopic studies^58^—as well as the feasibility of laboratory-based Se X-ray emission measurements.

## Supporting Information Available

Additional DFT and TDDFT calculations, fits of the experimental data, MO diagrams, sample input files, and *xyz* coordinates used in the calculations (PDF)
