# Supplementary material for: Selenium Valence-to-Core X-ray Emission Spectroscopy and Kβ HERFD X-ray Absorption Spectroscopy as Complementary Probes of Chemical and Electronic Structure
Source: Inorg Chem. 2022 Feb 3;61(6):2760–7. doi: 10.1021/acs.inorgchem.1c02802 (PMC8848279; doi:10.1021/acs.inorgchem.1c02802)
Supplement: Supplementary file 1 — ic1c02802_si_001.pdf [file ic1c02802_si_001.pdf]

Selenium Valence-to-Core X-ray Emission Spectroscopy and Kβ HERFD X-ray Absorption Spectroscopy as Complementary Probes of Chemical and Electronic Structure

Justin T. Henthorn[1] and Serena DeBeer[1,*]

[1]Max Planck Institute for Chemical Energy Conversion, Stiftstrasse 34-36, D-45470 Mülheim an der Ruhr, Germany

Supporting Information

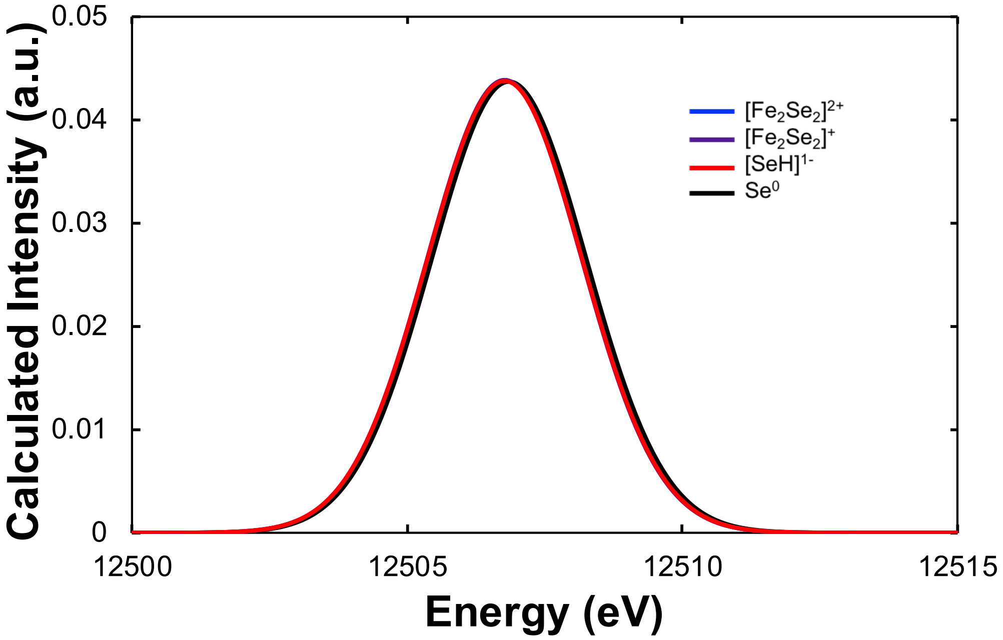

Figure S1. DFT calculated Se Kβ$_1$ XES spectra of $[Fe_2Se_2]^{2+}$ (blue), $[Fe_2Se_2]^{+}$ (purple), $[Et_4N][SeH]$ (red), and Se^0 (black). The maximum difference among the spectra is 0.1 eV (SeH^{1-} to Se^0).

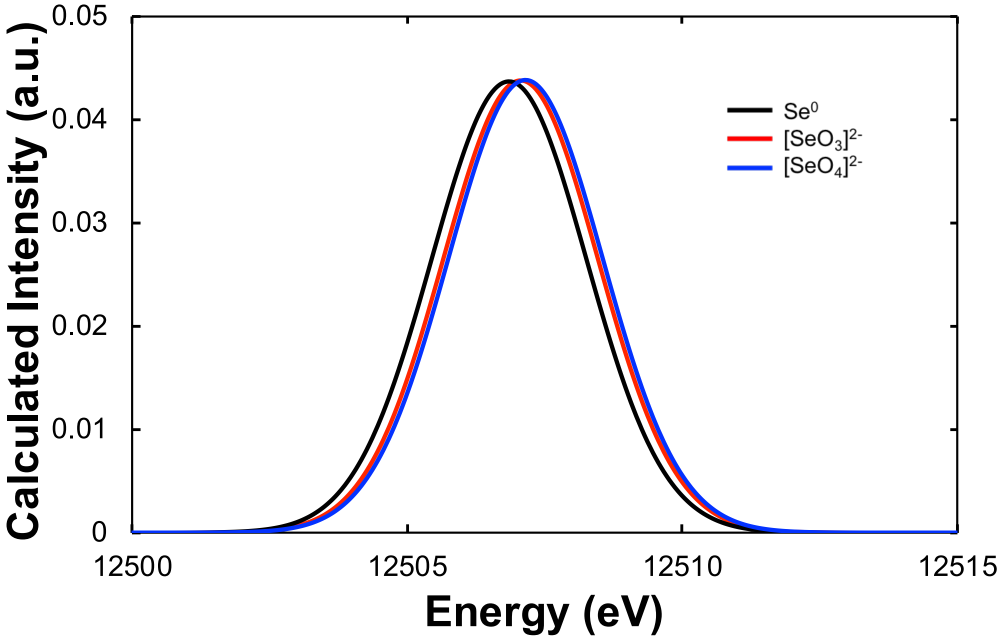

Figure S2. DFT calculated Se Kβ$_1$ (3p → 1s) XES spectra of Se^0 (black), $[SeO_3]2^-$ (red), and $[SeO_4]^{2-}$ (blue). The maximum difference is 0.3 eV (Se^0 to $[SeO_4]^{2-}$).

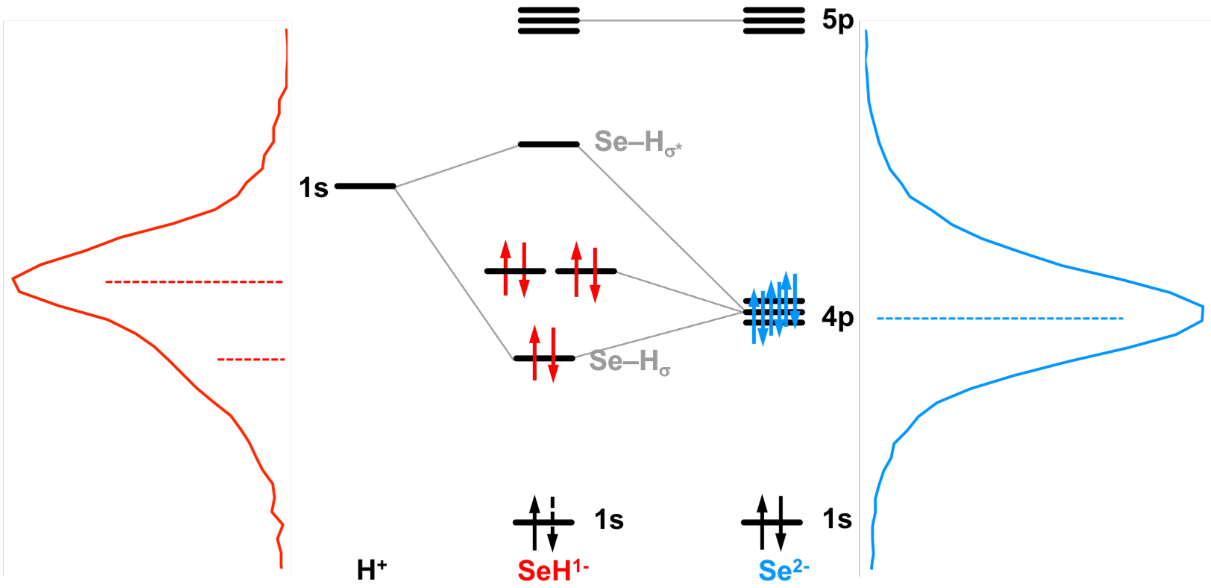

Figure S3. Qualitative MO diagram for $[SeH]^{1-}$ and comparison to the experimental Se VtC XES spectra of $[Et_4N][SeH]$ (left, red) and Li_2Se (teal, right). Vertical axis (energy) not to scale.

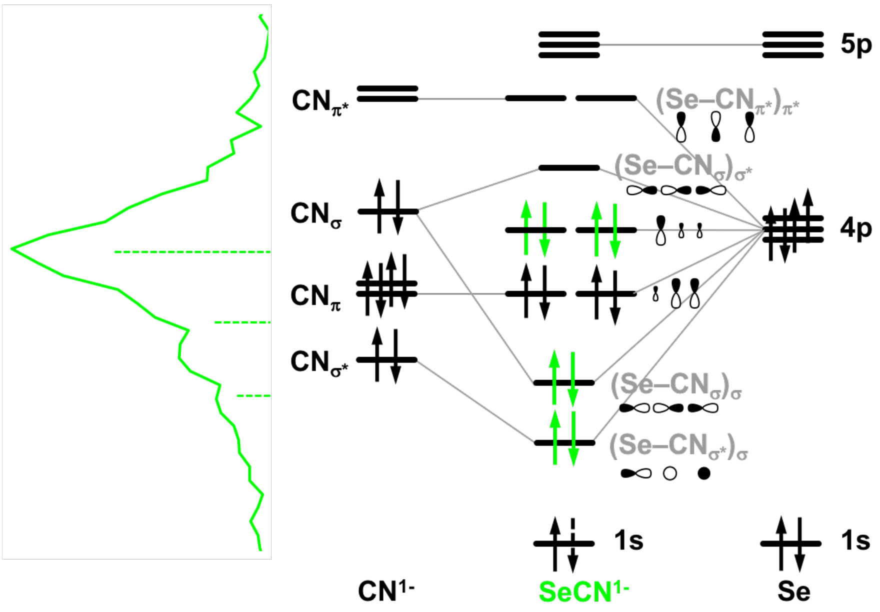

Figure S4. Qualitative MO diagram for $[SeCN]^{1-}$ and comparison to the experimental Se VtC XES spectrum of KSeCN (left, green). Vertical axis (energy) not to scale.

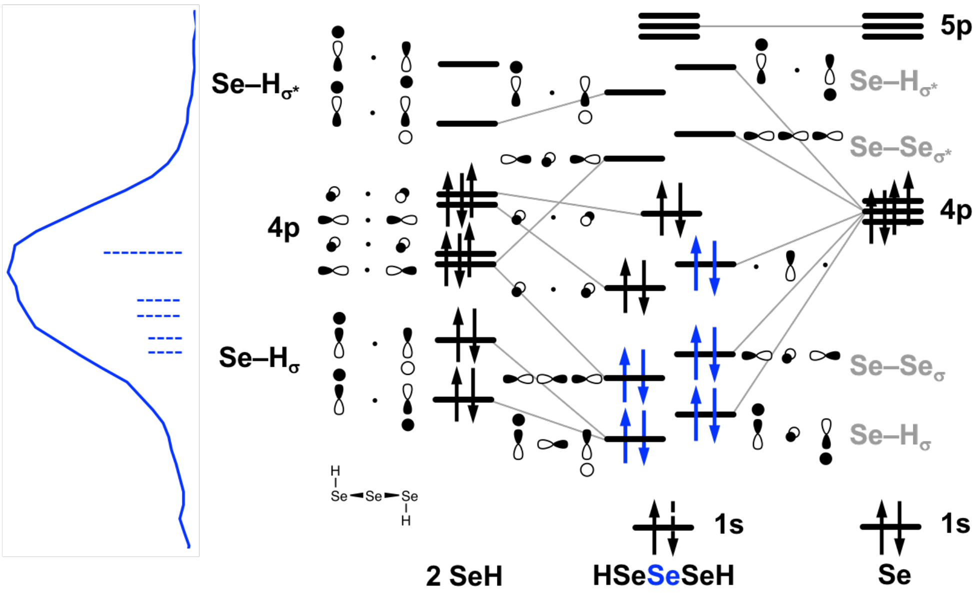

Figure S5. Qualitative MO diagram for HSeSeSeH and comparison to the experimental Se VtC XES spectrum of Se^0. Vertical axis (energy) not to scale.

Fits of Experimental Se VtC XES and HERFD XAS Spectra

The experimental data is shown in black. Individual edge Gaussians (for XAS) are shown as thin red lines and their sum is given as a thick red line. Individual pseudo-Voigt functions are shown as thin dark blue lines and their sum is given as a thick dark blue line. The difference between the fit and the experimental data is shown as a thin light blue line.

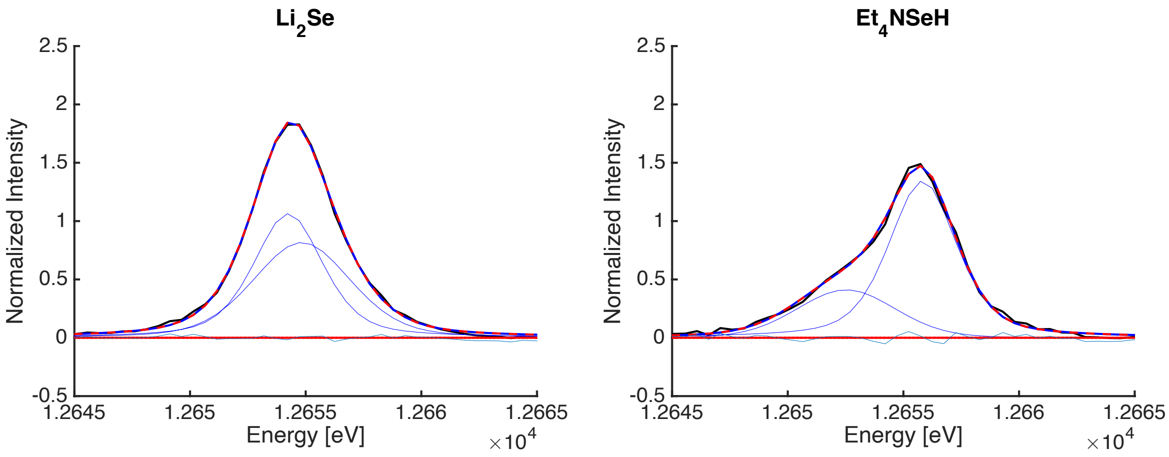

Figure S6. Fits of Se VtC XES for Li_2Se (left) and $[Et_4N][SeH]$ (right).

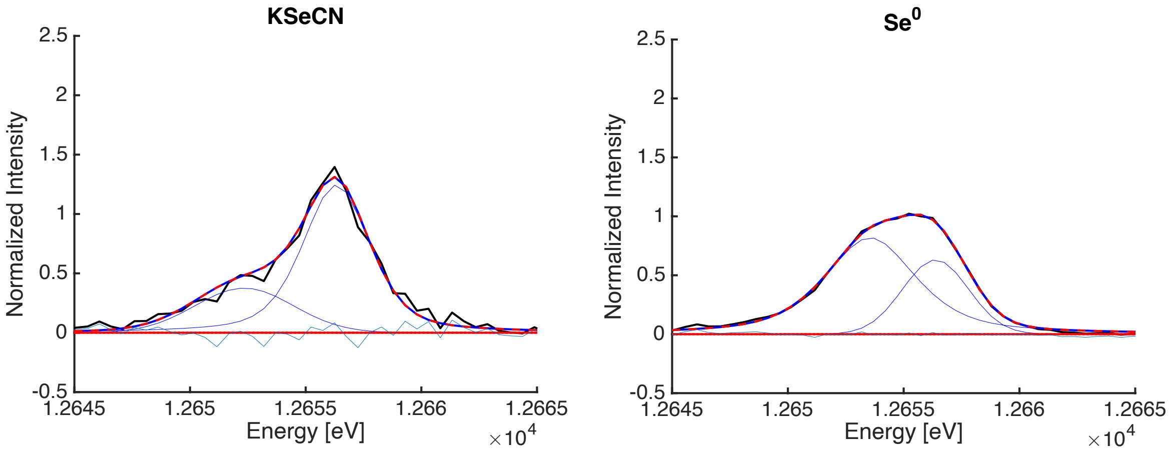

Figure S7. Fits of Se VtC XES for KSeCN (left) and gray elemental selenium (right).

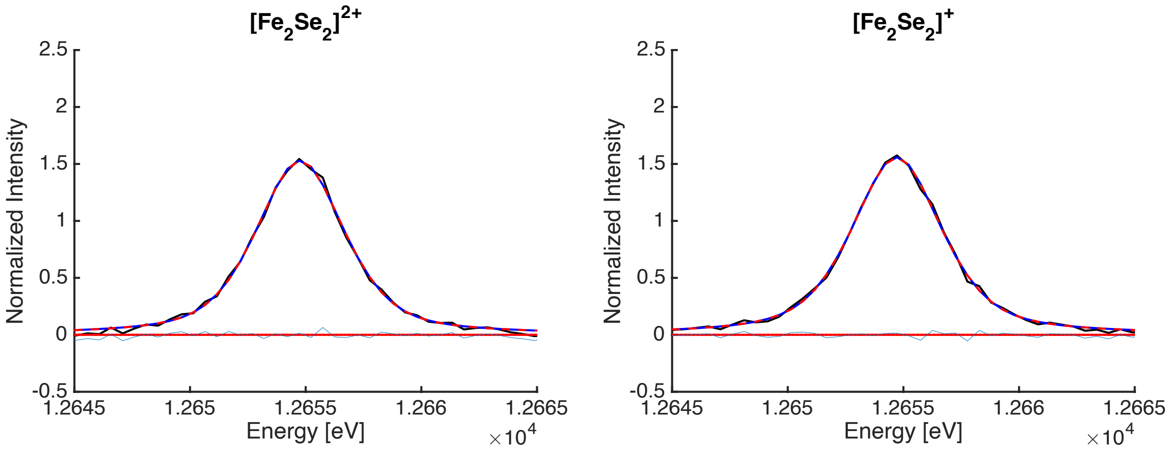

Figure S8. Fits of Se VtC XES for $L_2Fe_2Se_2$ (left) and $[K(THF)_6][L_2Fe_2Se_2]$•2THF (right).

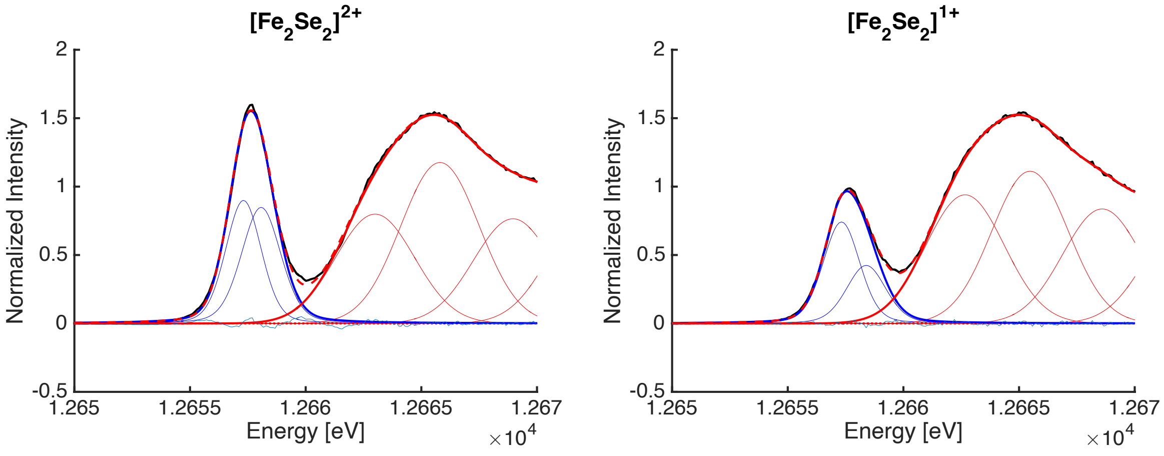

Figure S9. Fits of Se HERFD XAS for $L_2Fe_2Se_2$ (left) and $[K(THF)_6][L_2Fe_2Se_2]{\bullet}2THF$ (right).

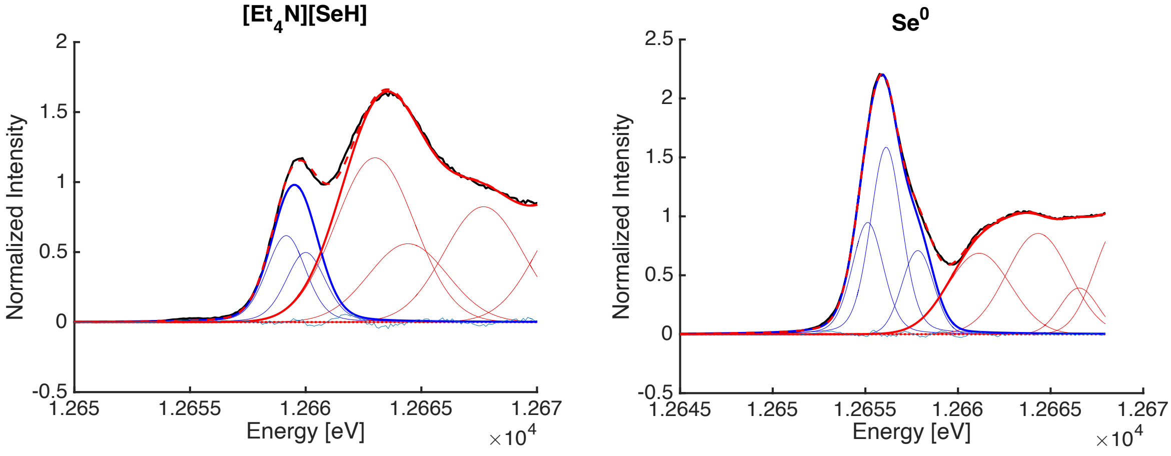

Figure S10. Fits of Se HERFD XAS for $[Et_4N][SeH]$ (left) and gray elemental selenium (right).

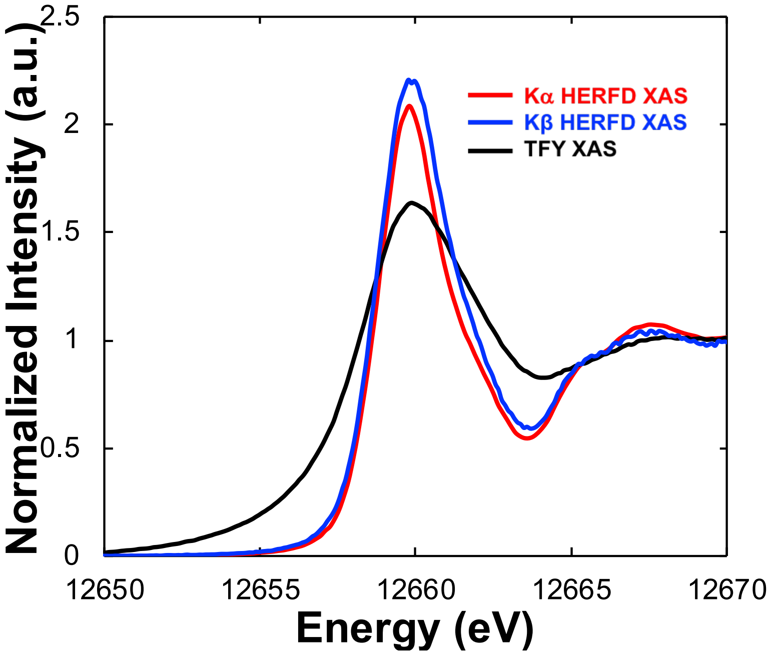

Figure S11. Comparison of Se Kα HERFD XAS (red) and Kβ HERFD XAS (blue) to total fluorescence yield (TFY) XAS (black) of gray elemental selenium. Se Kα HERFD XAS data measured at SSRL BL 6-2, see reference 1 for experimental conditions.

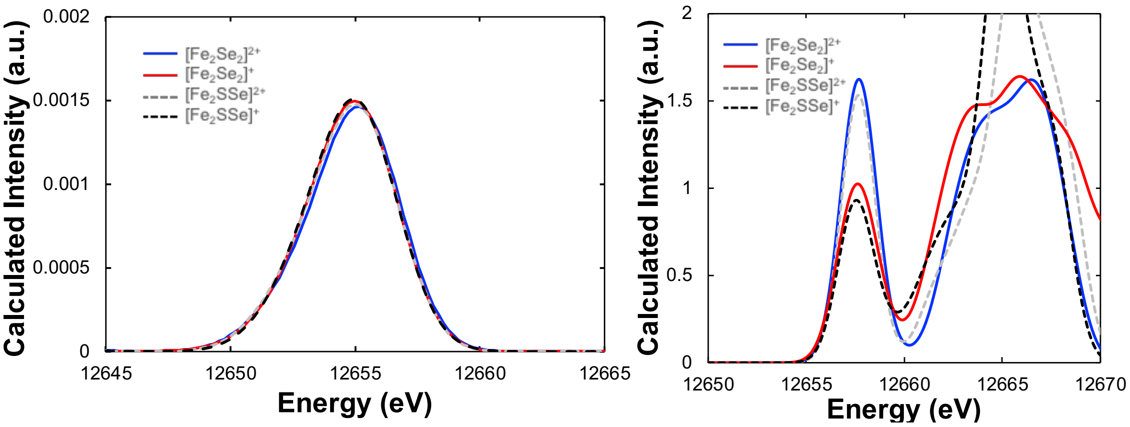

Figure S12. Comparison of calculated XES (left) and XAS (right) spectra of $[Fe_2Se_2]^{n+}$ (n=2, blue solid line; n=1, red solid line) and $[Fe_2SSe]^{n+}$ (n=2, gray dashed line; n=1, black dashed line) complexes.

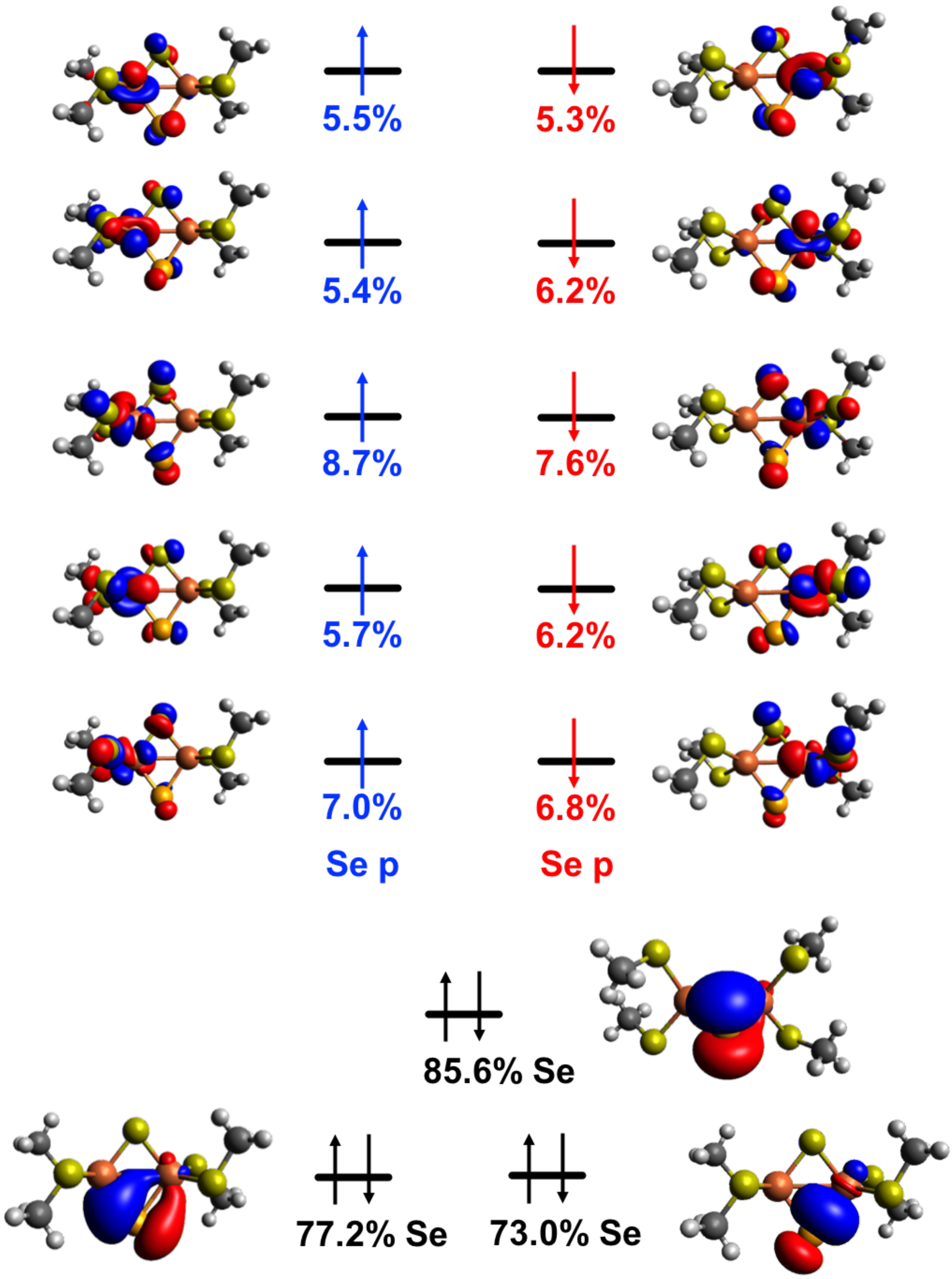

Figure S13. MO diagram of the $[Fe_2SSe]^{2+}$ complex with Se orbital contributions determined from the IAOIBO method. The nominal Fe–Se bonding and Se non-bonding orbitals are shown in black, while the spin-up Fe 3d orbitals are shown in blue and the spin-down Fe 3d orbitals are shown in red.

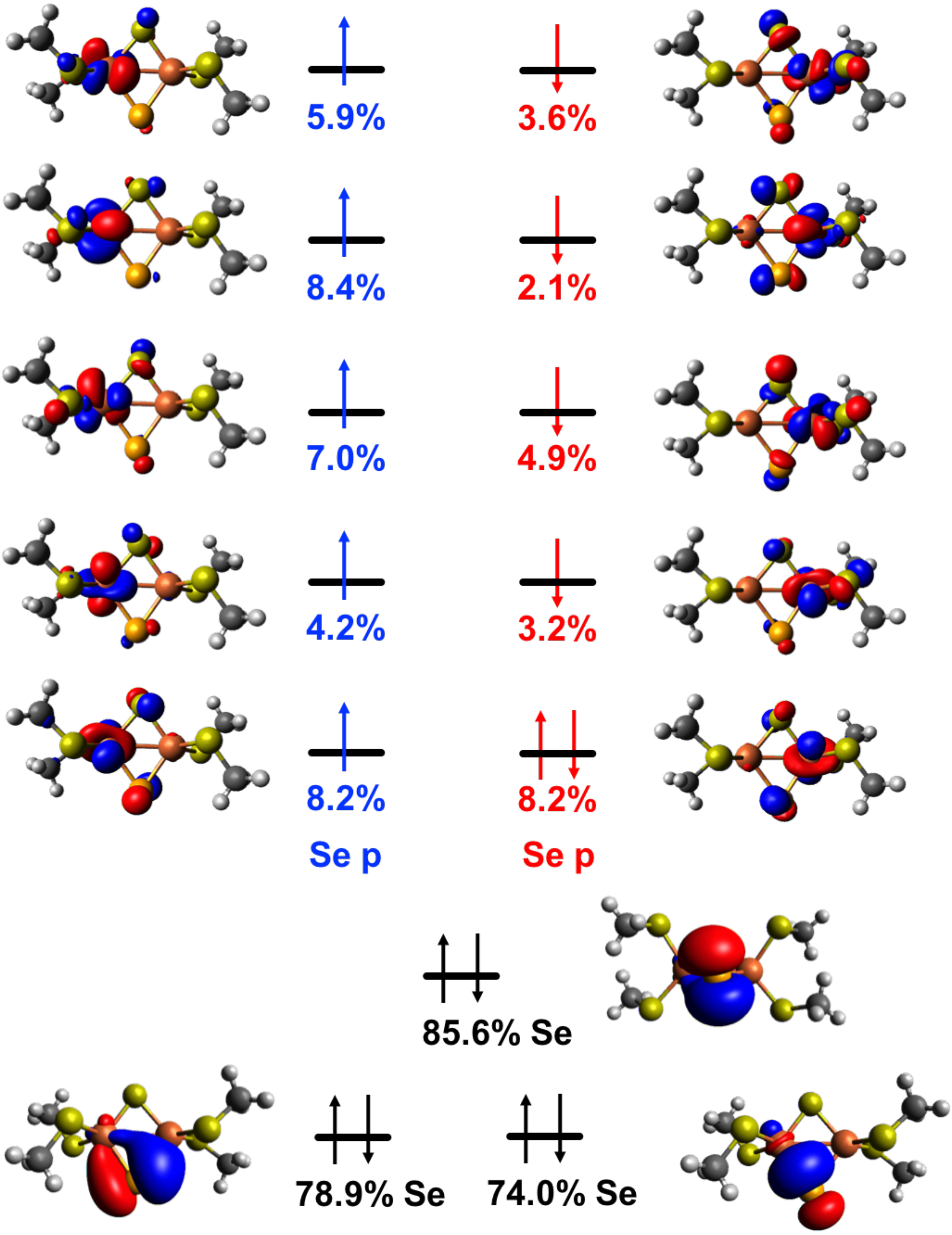

Figure S14. MO diagram of the $[Fe_2SSe]^+$ complex with Se orbital contributions determined from the IAOIBO method. The nominal Fe–Se bonding and Se non-bonding orbitals are shown in black, while the spin-up Fe 3d orbitals are shown in blue and the spin-down Fe 3d orbitals are shown in red.

Sample ORCA Input Files

ORCA input file for full geometry optimization (broken symmetry antiferromagnetic case):

```
! UKS TPSSh ZORA-def2-TZVP def2/J ZORA D3BJ CPCM RIJCOSX TightSCF
! Grid5 Finalgrid6 SlowConv
! Normalprint Opt

%maxcore 4000

%method
IntaccX 4.01,4.01,4.34 #Changing the 3 radial grids
GridX 1,1,2 #Changing the 3 angular grids
end

%basis
        newgto H "def2-SVP" end
        newgto C "def2-SVP" end
end

%scf
        MaxIter 500
        FlipSpin 1
        FinalMs 0
end

%pal nprocs 12
end

*xyzfile -2 11 Fe2SSe_S4Me4_diferric.xyz
```

ORCA input file for Se Valence-to-Core X-ray Emission Spectroscopy (VtC XES) calculation on broken symmetry solution:

```
! UKS TPSSh ZORA-def2-TZVP def2/J ZORA D3BJ CPCM RIJCOSX TightSCF
! Grid5 Finalgrid6 SlowConv
! Normalprint
! MORead
%moinp "Fe2SSe_S4Me4_diferric_BS_opt.gbw"

%method
IntaccX 4.01,4.01,4.34 #Changing the 3 radial grids
GridX 1,1,2 #Changing the 3 angular grids
end

%method SpecialGridAtoms 26
        SpecialGridIntAcc 7
end

%basis
```

```
	newgto H "def2-SVP" end
	newgto C "def2-SVP" end
end

%xes
	CoreOrb 0,0
	OrbOp 0,1
end

%pal nprocs 12
end

*xyzfile -2 1 Fe2SeS_S4Me4_diferric_BS_opt.xyz
```

ORCA TDDFT input file for Se X-ray Absorption Spectroscopy (XAS) calculation on broken symmetry solution:

```
! UKS TPSSh ZORA-def2-TZVP def2/J ZORA D3BJ CPCM RIJCOSX TightSCF
! Grid5 Finalgrid6 SlowConv
! Normalprint
! MORead
%moinp "Fe2SeS_S4Me4_diferric_BS_opt.gbw"

%method
IntaccX 4.01,4.01,4.34 #Changing the 3 radial grids
GridX 1,1,2 #Changing the 3 angular grids
end

%method SpecialGridAtoms 26
	SpecialGridIntAcc 7
end

%basis
	newgto H "def2-SVP" end
	newgto C "def2-SVP" end
end

%tddft	NRoots		100
	MaxDim		1000
	OrbWin[0]=0,0,-1,-1
	OrbWin[1]=0,0,-1,-1
	DoQuad		True
	TDA	True
end

%pal nprocs 12
end

*xyzfile -2 1 Fe2SeS_S4Me4_diferric_BS_opt.xyz
```

Structures used in XES and XAS Calculations

Structure of geometry optimized linear HSe(Se$_6$)SeH

Se	-4.34214761188564	-0.32700061973084	0.39128951344184
Se	-2.10383449131506	-0.89581688491581	0.02771030937913
Se	-1.13798957579542	0.93372767934378	-1.06266493830199
Se	-0.63036138444453	2.46358420591149	0.63037579383294
Se	-2.52950211323469	3.83056096999584	0.72345214337181
Se	-2.08648730325433	5.48794565646994	-0.86234461533757
Se	-1.08667253375428	7.23500229323561	0.33343213233594
Se	1.16482001100754	6.64960428712053	0.56753523764866
H	1.61376610341934	7.20132688427550	-0.72493228511516
H	-4.84627110074293	-0.77692447170608	-0.92019329125560

Coordinates of [Li$_8$Se]$^{6+}$

Se	0.00000	0.00000	0.00000
Li	1.49900	1.49900	1.49900
Li	1.49900	1.49900	-1.49900
Li	1.49900	-1.49900	1.49900
Li	-1.49900	1.49900	1.49900
Li	-1.49900	-1.49900	-1.49900
Li	-1.49900	-1.49900	1.49900
Li	-1.49900	1.49900	-1.49900
Li	1.49900	-1.49900	-1.49900

Structure of geometry optimized [SeH]$^{1-}$

Se	-3.27060563789805	0.10822000000000	-0.00562000000000
H	-1.79479436210195	0.10822000000000	-0.00562000000000

Structure of geometry optimized [SeCN]$^{1-}$

Se	-3.23783035301923	0.10445222396180	-0.00543243269858
C	-1.43433299439294	-0.02817926128594	0.00153911676017
N	-0.26243665258783	-0.11461296267586	0.00604331593841

Structure of geometry optimized [SeO$_3$]$^{2-}$

Se	-0.86814190882039	-1.74371962434688	0.72253130822477
O	0.27489135925285	-1.55133062230314	1.97498497629314
O	-0.38380188261441	-3.18339248825139	-0.05522611972422
O	-2.29872756781804	-2.18436726509858	1.54253983520631

Structure of geometry optimized [SeO$_4$]$^{2-}$

Se	-0.84852814773105	-1.86191221021971	0.77547455090344
O	-0.98472958209383	-0.58282698623620	-0.27157881798276
O	0.28289766248016	-1.50179534168153	1.93372192085547
O	-0.37620405760117	-3.21500331065463	-0.06027346045824
O	-2.31506587505410	-2.14528215120793	1.49805580668209

Structure of BS-optimized antiferromagnetic [Fe$_2$SSe(SMe)$_4$]$^{2-}$

Se	6.65165605451681	1.20443988110457	1.51191958227894
Fe	6.62126213557679	1.10803458507496	-0.83524489477437
Fe	6.61841544282433	-1.04098505329822	0.82270938710131

S	8.40929827317975	2.09889874905139	-1.86225571870264
S	4.72204867503204	2.20186531520454	-1.48682321699930
C	8.15500552004604	3.86322718625229	-1.46097099533139
C	4.76716422581919	1.99006068420248	-3.30126906292115
S	6.67708866838536	-1.02722756196675	-1.38678703430925
S	8.38198663015532	-2.06597926978077	1.86267301973334
S	4.70794911620605	-2.16576400403155	1.38799596902791
C	8.12242059804299	-3.82387720463578	1.43390666507200
C	4.76006583243387	-2.13317145643065	3.21341191548726
H	3.92372200790011	2.53631003547085	-3.74774958623226
H	4.68509456212069	0.92600729667985	-3.56477876837396
H	5.70937041314161	2.38591585325861	-3.70614882774441
H	8.89502815440675	4.46504289755475	-2.00771134896317
H	8.27776445713401	4.03537570641279	-0.38224499473685
H	7.14284190376439	4.17140574247528	-1.75911223728891
H	8.86271968792849	-4.43452197388804	1.97042731953984
H	8.24308310275720	-3.98167042575306	0.35284781661276
H	7.11085968951920	-4.13580130369330	1.73024294909169
H	3.93828231201415	-2.74796002286087	3.60786259971326
H	4.64684594239758	-1.10359580712397	3.58172060078277
H	5.71819712324857	-2.53548602722525	3.57207313950600

Structure of BS-optimized antiferromagnetic $[Fe_2SSe(SMe)_4]^{3-}$

Se	6.68064158118213	1.09707934896409	1.62537608010658
Fe	6.67550142584352	1.01620595834483	-0.82905229676118
Fe	6.64558297388937	-1.14342750533390	0.90555581760279
S	8.42064456234333	2.18210369078067	-1.81444146639284
S	4.71888659072013	2.02661531588005	-1.56067105952526
C	7.94057193856211	3.94326261482333	-1.68879263025401
C	4.90782818338400	2.31367216678491	-3.35751459338933
S	6.76754383810821	-1.22074791777266	-1.30138896809024
S	8.38997126775453	-2.19436824673490	2.02645022642007
S	4.72113987647841	-2.31234975584823	1.46831187801470
C	8.15895184261828	-3.94942329000806	1.57105492564483
C	4.74064023382476	-2.26501353307479	3.29422025519118
H	4.19127778624585	3.07423287073263	-3.70416109540465
H	4.72728188226131	1.38608312501466	-3.92291625589222
H	5.93029391825236	2.65904144141136	-3.57486696844610
H	8.53324574489393	4.55510639372281	-2.38581545597342
H	8.10725514269540	4.31787890809532	-0.66716479983669
H	6.87176241819959	4.05171845425773	-1.92721698606402
H	8.88724906041493	-4.56954497249649	2.11437382093059
H	8.30338188486508	-4.09345581873367	0.49027604533117
H	7.14136612933358	-4.27166004791263	1.83623625668882
H	3.91598534343269	-2.87593794789130	3.69064443783919
H	4.62464950079787	-1.23183629657236	3.65320757080751
H	5.69555126260358	-2.66070973527529	3.67015190404481

Structure of BS-optimized antiferromagnetic $[Fe_2S(SeH)(SMe)_4]^{1-}$

Se	6.66789609330480	1.38257247819670	1.62250352078363
Fe	6.56544693021873	1.19605147906292	-0.84958425203596

Fe	6.58659575919720	-0.99827653591079	0.87163574343524
S	8.44078059149423	2.08925145047484	-1.70164230400034
S	4.73068532948685	2.29392448390021	-1.54954180307443
C	8.19787306833675	3.88649663368175	-1.49395061144583
C	4.75591118345199	1.92800269708355	-3.34086594391798
S	6.61540654342375	-0.93861140918528	-1.31653450868915
S	8.32335262180699	-2.00951567601929	1.89467222288396
S	4.63854611968405	-1.95213276407321	1.44927379905749
C	8.08294961085657	-3.75731614452297	1.41462720210351
C	4.80360526530754	-2.27878327611104	3.23888822878088
H	3.93089984025523	2.47626469028132	-3.81573227550982
H	4.62579992943531	0.85108792923437	-3.51234588022899
H	5.70867648387844	2.25549604466045	-3.77864156594967
H	9.04339204501619	4.40834860122529	-1.96318373723170
H	8.15991113567799	4.14433263683234	-0.42671443180744
H	7.26019244734932	4.19665948459482	-1.97443597418571
H	8.79743905729548	-4.36947107403511	1.98162786704067
H	8.26354256861369	-3.89005441982855	0.33958099283786
H	7.06024828472334	-4.07826150106304	1.65554675544114
H	3.87411836772805	-2.74762011718927	3.59122551734143
H	4.96738627735578	-1.34103017694426	3.78645206598394
H	5.64751497465311	-2.95687169229158	3.42383364995667
H	5.21153947144842	1.43454617794587	1.84054572243061

Structure of BS-optimized antiferromagnetic $[Fe_2S(SeH)(SMe)_4]^{2-}$

Se	6.70252724061309	1.22915884201951	1.49518033664318
Fe	6.68731827768122	0.97638346482988	-1.00146642290458
Fe	6.61134493970764	-1.15977438663380	0.80062793804314
S	8.46863501962020	2.07861065842975	-1.92399814236756
S	4.72170100921333	2.04905264243936	-1.46905010634364
C	8.14041586990511	3.83200199769744	-1.51565835591458
C	4.69968978456993	2.18596197075652	-3.29393299248313
S	6.75834578681358	-1.23747490696592	-1.37837651845642
S	8.33471595513359	-2.06301413609184	2.00949651015863
S	4.64108732671561	-2.14139144003254	1.36353910141527
C	8.25150317445718	-3.83213550266841	1.55692419999742
C	4.66432392454138	-2.17448488447305	3.18988544387491
H	3.87549731650454	2.84035874157660	-3.61585993336459
H	4.56250147237404	1.19751590150204	-3.75800267583832
H	5.65022811960902	2.61020960913430	-3.65083470077153
H	8.83726088001544	4.47776412992065	-2.06992876123417
H	8.27197264476518	4.00829958579111	-0.43810922439071
H	7.10807948049944	4.09064707337625	-1.79136262094682
H	9.01303841331452	-4.37945286202339	2.13015890460069
H	8.44142673608413	-3.96896991790877	0.48331649619808
H	7.25888226250375	-4.23517969028799	1.80211301311914
H	3.84155749713191	-2.80952082148453	3.54812949647879
H	4.53426554857477	-1.15971621588538	3.59164160458561
H	5.62088570835646	-2.58032463185971	3.54742405249348
H	5.26250561129453	1.39056477884192	1.77538335740774

Reference

1. Henthorn, J. T.; Arias, R. J.; Koroidov, S.; Kroll, T.; Sokaras, D.; Bergmann, U.; Rees, D. C.; DeBeer, S., Localized Electronic Structure of Nitrogenase FeMoco Revealed by Selenium K-Edge High Resolution X-ray Absorption Spectroscopy. *Journal of the American Chemical Society* **2019,** *141* (34), 13676-13688.
